# L-arginine supplementation abrogates hypoxia-induced virulence of *Staphylococcus aureus* in a murine diabetic pressure wound model

**DOI:** 10.1128/msphere.00774-23

**Published:** 2024-03-01

**Authors:** Carol L. Baker, Keun Seok Seo, Nogi Park, Jaime K. Rutter, Justin A. Thornton, Stephen B. Pruett, Joo Youn Park

**Affiliations:** 1 Department of Comparative Biomedical Sciences, College of Veterinary Medicine, Mississippi State University, Mississippi State, Mississippi, USA; 2 Department of Biological Sciences, College of Arts and Sciences, Mississippi State University, Mississippi State, Mississippi, USA; University of Kentucky College of Medicine, Lexington, USA

**Keywords:** hypoxia, virulence, *Staphylococcus aureus*, L-arginine, diabetic foot ulcers

## Abstract

Diabetic foot ulcers (DFUs) are the most common complications of diabetes resulting from hyperglycemia leading to ischemic hypoxic tissue and nerve damage. *Staphylococcus aureus* is the most frequently isolated bacteria from DFUs and causes severe necrotic infections leading to amputations with a poor 5-year survival rate. However, very little is known about the mechanisms by which *S. aureus* dominantly colonizes and causes severe disease in DFUs. Herein, we utilized a pressure wound model in diabetic TALLYHO/JngJ mice to reproduce ischemic hypoxic tissue damage seen in DFUs and demonstrated that anaerobic fermentative growth of *S. aureus* significantly increased the virulence and the severity of disease by activating two-component regulatory systems leading to expression of virulence factors. Our *in vitro* studies showed that supplementation of nitrate as a terminal electron acceptor promotes anaerobic respiration and suppresses the expression of *S. aureus* virulence factors through inactivation of two-component regulatory systems, suggesting potential therapeutic benefits by promoting anaerobic nitrate respiration. Our *in vivo* studies revealed that dietary supplementation of L-arginine (L-Arg) significantly attenuated the severity of disease caused by *S. aureus* in the pressure wound model by providing nitrate. Collectively, these findings highlight the importance of anaerobic fermentative growth in *S. aureus* pathogenesis and the potential of dietary L-Arg supplementation as a therapeutic to prevent severe *S. aureus* infection in DFUs.

**IMPORTANCE:**

*S. aureus* is the most common cause of infection in DFUs, often resulting in lower-extremity amputation with a distressingly poor 5-year survival rate. Treatment for *S. aureus* infections has largely remained unchanged for decades and involves tissue debridement with antibiotic therapy. With high levels of conservative treatment failure, recurrence of ulcers, and antibiotic resistance, a new approach is necessary to prevent lower-extremity amputations. Nutritional aspects of DFU treatment have largely been overlooked as there has been contradictory clinical trial evidence, but very few *in vitro* and *in vivo* modelings of nutritional treatment studies have been performed. Here we demonstrate that dietary supplementation of L-Arg in a diabetic mouse model significantly reduced duration and severity of disease caused by *S. aureus*. These findings suggest that L-Arg supplementation could be useful as a potential preventive measure against severe *S. aureus* infections in DFUs.

## INTRODUCTION

Type 2 diabetes mellitus (T2DM) is a worldwide health care crisis affecting an estimated 422 million people and is the direct cause of death in about 1.5 million people each year (1). Hyperglycemia caused by T2DM causes a multitude of issues in the body that lead to serious damage to the heart, blood vessels, eyes, kidneys, nerves, and skin of the diabetic patient. Diabetic foot ulcer (DFU) is the most common complication of T2DM (2, 3). Approximately, 25% of all T2DM patients will develop a DFU in their lifetime, with over 65% of DFUs reoccurring within 5 years post-healing (4, 5). It is estimated that 50% of DFUs will become infected, resulting in a 155-fold increased risk of amputation compared to sterile DFUs (6, 7). *Staphylococcus aureus* is the most common pathogen isolated from DFU wounds, accounting for 50%–90% of infected DFUs (8). Moreover, *S. aureus-*infected DFUs are particularly difficult to treat due to antibiotic resistance, biofilm formation, and severe osteomyelitis, leading to salvage amputations with a poor 5-year survival rate (9, 10).

DFUs are formed due to hyperglycemia causing peripheral neuropathy, decreased blood flow, hypoxia, and altered foot architecture that causes pressure being put on an inappropriate anatomical part of the foot (11). This repeated pressure and reperfusion cause injury to the underlying skin that forms ischemic ulcers (12). These comorbidities make metabolic conditions in DFU tissues vary from non-diabetic ulcers in several ways. Firstly, diabetes is a complex metabolic disorder that elevates not only glucose but also other sugars found in the body, especially intracellular accumulation of glucose-6-phosphate (G6P). This is due to hyperglycemic deactivation of intracellular glucose-6-phosphate dehydrogenase (G6PD) that is necessary to convert G6P to 6-phosphogluconate (13, 14). Previous studies demonstrated that *S. aureus* possesses expanded glucose import capability for enhanced glycolytic flux and increased virulence under hyperglycemic conditions (15, 16). We demonstrated that G6P was highly abundant in skin adipose tissues of diabetic TALLYHO/JngJ mice, mirroring human T2DM skin adipose tissue concentrations. Subcutaneous infection with *S. aureus* showed significantly higher expression of staphylococcal cytotoxins, greater tissue necrosis, and bacterial burden compared to non-diabetic mice. A deletion of UhpT, a G6P transporter, significantly attenuated pathogenicity of *S. aureus* in diabetic TALLYHO/JngJ mice (17). These results indicated that metabolism of sugars highly elevated in T2DM, such as glucose and G6P, plays an important role in the pathogenesis of *S. aureus* within DFUs.

DFU tissue is in a state of chronic hypoxia resulting from hyperglycemic deactivation of the hypoxia inducible factor-1alpha (HIF-1α), which plays a central role in maintaining oxygen homeostasis in the body (18). This leads to the inability of tissues to sense oxygen deprivation, resulting in chronic hypoxia. Without HIF-1α, vascular endothelial growth factor is downregulated, while vascular endothelia growth factor repressor is upregulated. This decreases angiogenesis, which further decreases the oxygen supply to the tissues (18). Infections at DFU further exacerbate diabetic ischemia due to the massive oxidative bursts and glucose consumption by immune cells (19). These findings suggest that *S. aureus* must adapt to hypoxic conditions present in DFUs for successful pathogenesis.

*S. aureus* can survive hypoxic conditions by switching metabolic flux between fermentative growth and anaerobic nitrate respiration, depending on the availability of a terminal electron acceptor (TEA) such as nitrate (NO^−^_3_) and nitrite (NO^−^_2_) (20). The lack of a TEA impairs anaerobic respiration and accumulated reduced menaquinone induces activation of SrrAB two-component regulatory system (TCRS). This leads to fermentative growth of *S. aureus* that increases biofilm formation and expression of staphylococcal cytotoxins (21, 22). However, most of these studies have been performed *in vitro*, and the effects of diabetic metabolic conditions such as hypoxia and multiple elevated sugar levels on the *in vivo* pathogenesis of *S. aureus* have not been tested.

In this study, we utilized a pressure wound model in diabetic TALLYHO/JngJ to investigate the effect of diabetic metabolic conditions on the virulence of *S. aureus*. Our results show that hypoxic conditions, induced by pressure wounds in diabetic TALLYHO/JngJ mice, significantly increase the severity of disease caused by *S. aureus* infection.

## RESULTS

### Ischemic hypoxic wounds in diabetic mice significantly increased the virulence of *S. aureus* and delayed wound healing

**Fig 1:**
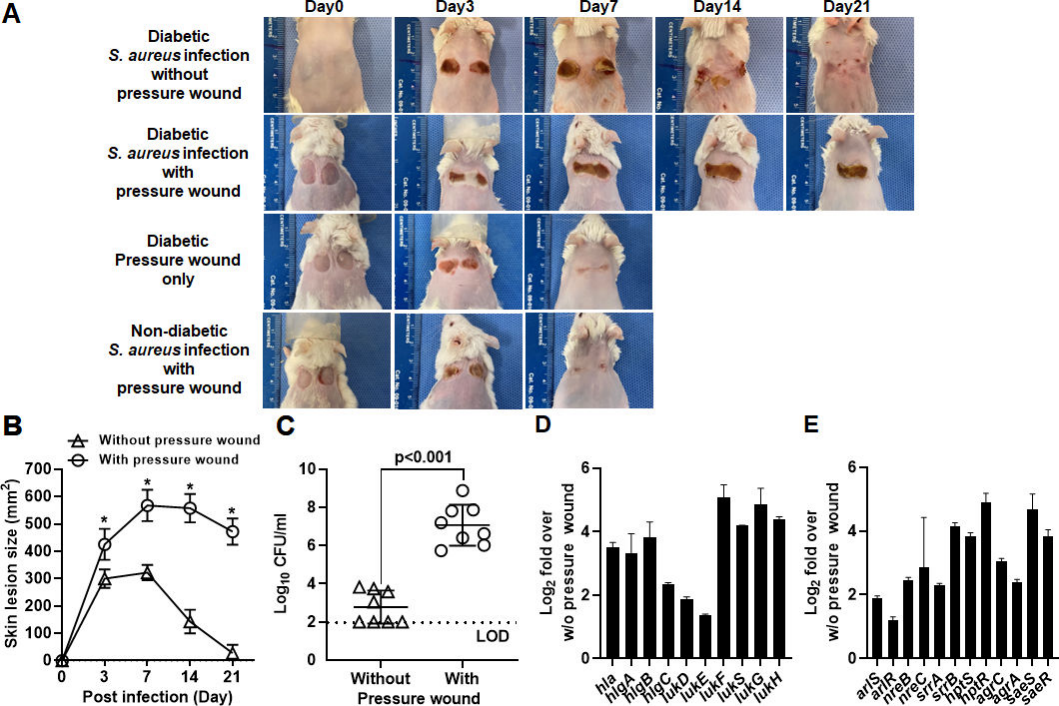
Hypoxic condition induced by a pressure wound model significantly increases the virulence of *S. aureus* in diabetic TALLYHO/JngJ mice. (**A**) Diabetic TALLYHO/JngJ mice (*n* = 8 mice/group) or non-diabetic SWR/J control mice (*n* = 8 mice/group) were subcutaneously infected with 1 × 10^7^ of *S. aureus* LAC strain in the ischemic pressure wound. As comparisons, diabetic TALLYHO/JngJ mice were subcutaneously infected without ischemic pressure wound or were not infected with ischemic pressure wound. Progress of wound healing was monitored daily for 21 days. (**B**) Analysis of areas of wound measured using the SilhouetteStar camera. Representative images from three independent experiments are shown. (**C**) Analysis of bacterial burden of infected site at day 21 post-infection. (**D and E**) Quantitative real-time PCR analysis of (**D**) virulence factors and (**E**) TCRS at the infection site with the pressure wound compared to that without pressure wound at day 7 post-infection. Data are presented as the mean ± standard deviation of three independent experiments. Statistical significance was evaluated by Student’s *t*-test. *P < 0.01. LOD, limit of detection.

DFUs initially form by repeated pressure trauma and reperfusion that lead to calluses and eventual ulcer formation under the callus. This break in the skin barrier leaves the foot vulnerable to infection (8). To recapitulate this repeated trauma and reperfusion injury seen in clinical DFUs that leads to the classic ischemic hypoxic wound conditions, we induced ischemic pressure wounds in diabetic TALLYHO/JngJ mice or non-diabetic SWR/J control mice by the dorsal application and subsequent removal of two round ceramic magnets for two 12-hour on/off cycles as previously described (23). We then subcutaneously infected ischemic pressure wounds with 1 × 10^7^ CFU of *S. aureus* LAC strain, which is common in skin and soft-tissue infections (24, 25). Diabetic mice with infected pressure wounds developed significantly larger-tissue necrosis (Fig. 1A and B) and higher bacterial burden at day 21 post-infection (Fig. 1C) than infections without pressure wounds. Conversely, in non-diabetic mice, infected pressure wounds were healed within 7 days without causing severe tissue necrosis (Fig. 1A). Notably, in diabetic mice, *S. aureus* infections without pressure wounds were completely healed within 21 days post-infection, while infections at pressure wound remained unhealed (Fig. 1A). Control sterile pressure wounds were healed within 7 days. These results indicated that hypoxic conditions in diabetic tissues significantly increased the virulence of *S. aureus*.

To understand how *S. aureus* significantly increased severity and prolonged the duration of infected ischemic pressure wounds in diabetic mice, we analyzed transcription of genes encoding staphylococcal cytotoxins (*hla*, *hlgA*, *hlgB*, *hlgC*, *lukD*, *lukE*, *lukF*, *lukG*, *lukH*, and *lukS*) and TCRS using quantitative real-time PCR (qRT-PCR) on infected tissue homogenates at day 7 post-infection. Results showed that transcription of staphylococcal cytotoxins was highly increased in the infections with pressure wounds compared to those without (Fig. 1D). Concurrently, transcription of TCRS involved in oxygen stress (*arlRS*, *nreBC*, and *srrAB*), nutrients (*hptRS*), and expression of virulence factors (*agrAC* and *saeRS*) was increased from two- to fivefold in the infections with pressure wounds, compared to those without (Fig. 1E). These results suggest that the hypoxic conditions in diabetic pressure wounds significantly increased the severity of the infection caused by *S. aureus* through transcriptional activation of TCRS, leading to increased expression of staphylococcal virulence factors.

### *In vitro S. aureus* growth under diabetic metabolic conditions increased expression of *S. aureus* virulence factors

**Fig 2:**
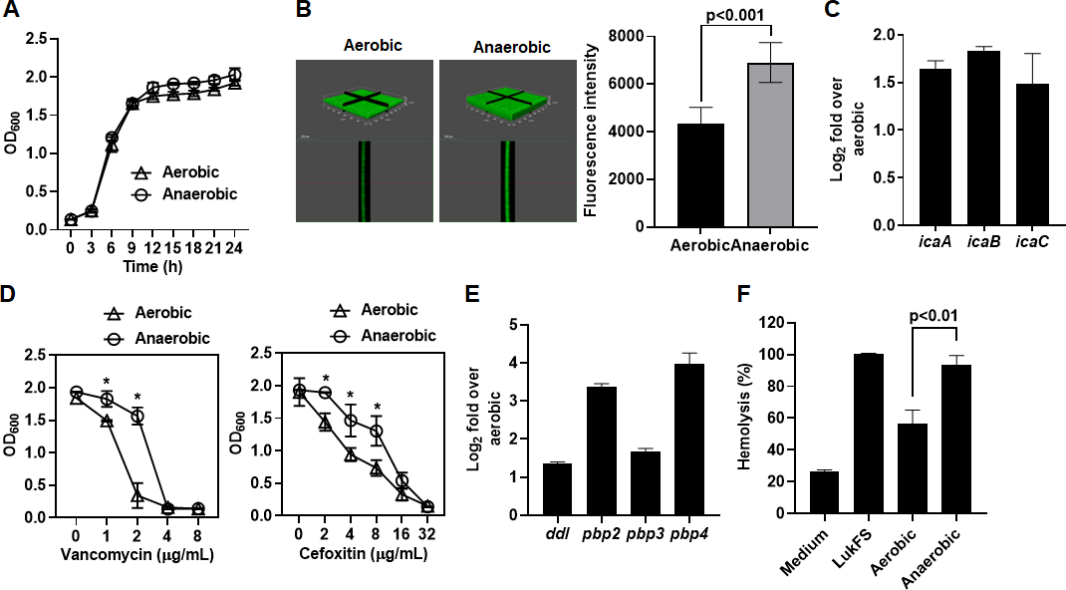
*In vitro S. aureus* growth under hypoxic and elevated sugar conditions increases expression of *S. aureus* virulence factors. (**A**) Aerobic or anaerobic growth of *S. aureus* LAC strain in BHI-G6P broth was determined by measuring the OD_600_ for 24 hours. (**B**) *S. aureus* LAC strain chromosomally integrated with the *gfp* gene aerobically or anaerobically was cultured in BHI-G6P broth for 24 hours, and biofilm formation was analyzed using a confocal microscopy by measuring the mean fluorescence intensity (*n* = 8). Representative images from three independent experiments are shown. (**C**) qRT- PCR analysis of the genes related to biofilm formation in anaerobic conditions compared to aerobic conditions. (**D**) Growth analysis of *S. aureus* LAC strain aerobically or anaerobically cultured in BHI-G6P broth supplemented with the indicated amount of vancomycin and cefoxitin was determined by measuring the OD_600_ after 24 hours. (**E**) qRT- PCR analysis of the genes related to cell wall synthesis in anaerobic conditions compared to aerobic conditions. (**F**) Human RBC was incubated with supernatants from *S. aureus* LAC strain aerobically or anaerobically cultured in BHI-G6P broth at 37°C for 1 hour, positive control (LukF and LukS, 1 µg of each) or negative control (medium). The OD_540_ value relative to the positive control was calculated to assess hemolytic activity. Data are presented as the mean ± standard deviation of three independent experiments. Statistical significance was evaluated by Student’s *t*-test. *P < 0.01.

To gain better insight into mechanisms by which *S. aureus* caused more severe infection within diabetic hypoxic pressure wounds, we performed *in vitro* studies using brain heart infusion broth supplemented with 0.2% (wt/vol) G6P (BHI-G6P). We selected this condition as brain heart infusion (BHI) already contains 0.2% glucose, which is equivalent to glucose levels seen in unregulated T2DM patients (200 mg/dL). We supplemented the BHI with 0.2% G6P, as we have previously demonstrated its important role in the pathogenesis of *S. aureus* in diabetic TALLYHO/JngJ mice (17). *S. aureus* LAC strain was anaerobically or aerobically cultured in BHI-G6P, and expression and transcription of virulence factors, including biofilm formation, antibiotic resistance, and cytotoxin production, were compared. While the growth of *S. aureus* was not affected by oxygen levels (Fig. 2A), anaerobic conditions significantly increased biofilm formation (Fig. 2B) with concurrent transcriptional increases of genes related to biofilm formation (*icaA*, *icaB*, and *icaC*) (Fig. 2C), compared to aerobic growth. Anaerobic growth of *S. aureus* significantly increased resistance to vancomycin and cefoxitin (Fig. 2D) with concurrent transcriptional increase of genes related to cell wall synthesis/antibiotic resistance (*ddl*, *pbp2*, *pbp3*, and *pbp4*) (Fig. 2E). Finally, the culture supernatants from anaerobic growth showed significantly greater lysis of human red blood cell (RBC) compared to aerobic growth, indicating increased expression of staphylococcal cytotoxins (Fig. 2F). Combined, these results indicated that hypoxia and elevated sugar levels significantly increased the expression of *S. aureus* virulence factors.

### Induction of anaerobic respiration by supplementing sodium nitrate as a TEA caused inactivation of TCRS, leading to decreased expression of *S. aureus* virulence factors in hypoxic conditions

**Fig 3:**
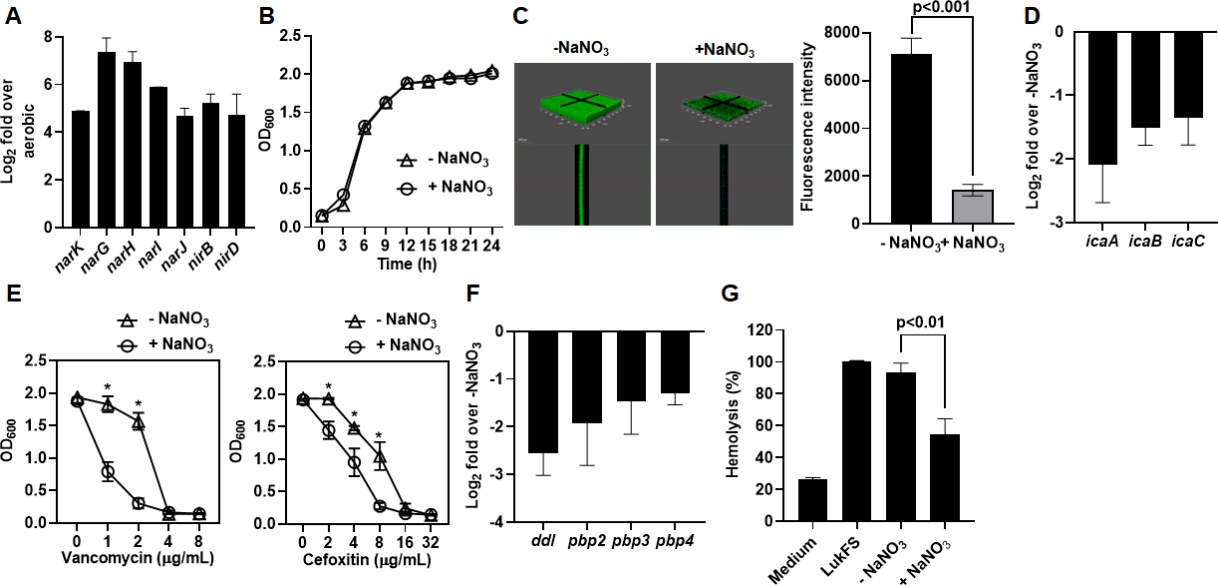
Supplementation of sodium nitrate suppresses expression of *S. aureus* virulence factors under hypoxic conditions. (**A**) qRT-PCR analysis of the gene related to anaerobic nitrate respiration in *S. aureus* LAC strain anaerobically or aerobically cultured in BHI-G6P broth for 24 hours. (**B**) Growth of *S. aureus* LAC strain cultured in BHI-G6P broth supplemented with or without 2-mM NaNO_3_ was determined by measuring the OD_600_ at the indicated time points for 24 hours. (**C**) Confocal microscopy images and the mean fluorescence intensity of biofilm formation by *S. aureus* LAC strain chromosomally integrated with the *gfp* gene anaerobically cultured in BHI-G6P supplemented with or without 2 mM NaNO_3_ for 24 hours. (**D**) qRT- PCR analysis of the genes related to biofilm formation in *S. aureus* LAC strain anaerobically cultured in BHI-G6P broth supplemented with or without 2 mM NaNO_3_. (**E**) Growth analysis of *S. aureus* LAC strain anaerobically cultured in BHI-G6P broth supplemented with or without 2 mM NaNO_3_ and the indicated amount of vancomycin and cefoxitin was determined by measuring the OD_600_ after 24 hours. (**F**) qRT- PCR analysis of the genes related to cell wall synthesis in *S. aureus* LAC strain anaerobically cultured in BHI-G6P broth supplemented with or without 2 mM NaNO_3_. (**G**) Human RBC was incubated with supernatants from *S. aureus* LAC strain anaerobically cultured in BHI-G6P broth supplemented with or without 2 mM NaNO_3_ for 1 hour, positive control (LukF and LukS, 1 µg of each) or negative control (medium). The OD_540_ value relative to the positive control was calculated to assess hemolytic activity. Data are presented as the mean ± standard deviation of three independent experiments. Statistical significance was evaluated by Student’s *t*-test. *P < 0.01.

**Fig 4:**
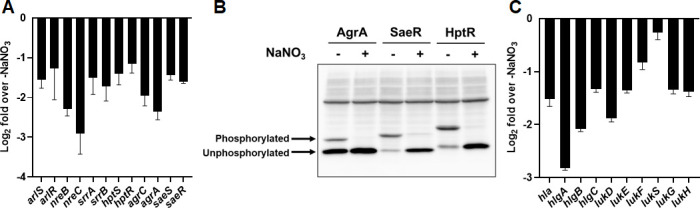
Sodium nitrate induces transcriptional decrease of TCRS and dephosphorylation of response regulatory leading to decreased expression of *S. aureus* virulence factors. (**A**) qRT-PCR analysis of global regulatory TCRS in *S. aureus* LAC strain anaerobically cultured for 24 hours in BHI-G6P broth supplemented with or without 2 mM NaNO_3_. (**B**) Phosphorylation of Agr, SaeR, or HptR fused with 3× FLAG in *S. aureus* LAC strain anaerobically cultured in BHI-G6P broth with or without supplementation of 2 mM NaNO_3_ for 24 hours was analyzed using a Phos-tag PAGE, followed by Western blot using an anti-FLAG mAb. Phos-tag bound phosphorylated protein has a slower migration rate than unbound non-phosphorylated protein, resulting in detectable separation of phosphorylated protein from non-phosphorylated protein. Representative images from two independent experiments are shown. (**C**) qRT- PCR analysis of staphylococcal cytotoxin genes in *S. aureus* LAC strain anaerobically cultured for 24 hours in BHI-G6P broth supplemented with or without 2 mM NaNO_3_ (*n* = 3).

To analyze the metabolic status of *S. aureus* growth under diabetic metabolic conditions, we determined the transcription of genes related to anaerobic nitrate respiration using qRT-PCR without any nitrate supplementation (Fig. 3A). Transcription of *narK*, which is required for uptake of nitrate, was increased by 29-fold. Transcription of *nar* (*G*/*H*/*I*/*J*) required for membrane-bound nitrate reductase reducing nitrate to nitrite was increased by 18- to 210-fold. Transcription of *nirB*/*D,* required for reducing nitrite to ammonia, increased by 16- to 64-fold. These results indicated that *S. aureus* growth under diabetic metabolic conditions increases transcription of genes related to anaerobic nitrate respiration even in the absence of nitrate. These results were somewhat surprising because transcriptional increase of SrrAB, NreBC, and cytotoxin production is a characteristics of *S. aureus* fermentative growth under anaerobic conditions (20, 26, 27). Thus, we expected transcriptional decreases of genes related to anaerobic respiration. This suggests that *S. aureus* spp. grown under diabetic metabolic conditions prefer anaerobic nitrate respiration but are forced to fermentative growth due to the absence of a TEA. To test this possibility, *S. aureus* LAC strain was anaerobically cultured in BHI-G6P broth with or without supplementation of 2-mM sodium nitrate as a TEA as previously demonstrated (20), and expression of virulence factors was compared. While not affecting the growth of *S. aureus* (Fig. 3B), the supplementation of sodium nitrate significantly reduced biofilm formation (Fig. 3C), transcription of genes related to biofilm formation (Fig. 3C), resistance to antibiotics (Fig. 3E), transcription of genes related to cell wall synthesis and antibiotic resistance (Fig. 3F), and production of staphylococcal cytotoxins (Fig. 3G). Decreased expression of staphylococcal virulence factors by supplementation of nitrate was accompanied with transcriptional decrease of global regulatory TCRS network (*arlRS*, *nreBC*, *srrAB*, *agrAC*, *hptRS*, and *saeRS*) (Fig. 4A). Since gene regulation by TCRS is mediated not only by its transcriptional level but also by the phosphorylation status of the response regulator, we analyzed the phosphorylation status of response regulators of the TCRS networks (AgrA, SaeR, and HptR) using mobility shift of 3× FLAG-tagged AgrA, SaeR, or HptR in a Phos-tag PAGE detected by Western blot using anti-FLAG M2 monoclonal antibody. This is possible because Phos-tag [1,3-bis(bis(pyridin-2-ylmethyl)amino)propan-2-olato diMn(II) complex] binds to phosphomonoester dianions (ROPO_3_^2−^) and thus retards the migration of phosphorylated proteins. *S. aureus* LAC strain anaerobically cultured in BHI-G6P broth showed higher intensity of slow-migrating bands than fast-migrating bands, while supplementation of sodium nitrate showed higher intensity of fast-migrating bands than slow-migrating bands. These results indicate that anaerobic culture of *S. aureus* LAC strain in BHI-G6P broth induced phosphorylation of response regulators which are dephosphorylated by supplementation of sodium nitrate (Fig. 4B). Consistently, transcription of staphylococcal cytotoxin genes regulated by AgrA and SaeR was highly decreased (Fig. 4C).

### Dietary L-Arg supplementation significantly decreased the severity of disease caused by *S. aureus* infection in the pressure wounds in diabetic TALLYHO/JngJ mice

Despite promising *in vitro* results above, care needs to be taken for the direct *in vivo* use of sodium nitrate as a therapeutic as nitrate can cause side effects such as acute acquired methemoglobinemia, hypotension, and angina-like pain. Furthermore, it has been classified as a group 2A carcinogen known as “probably carcinogenic to humans” by the World Health Organization’s International Agency for Research on Cancer (28).

**Fig 5:**
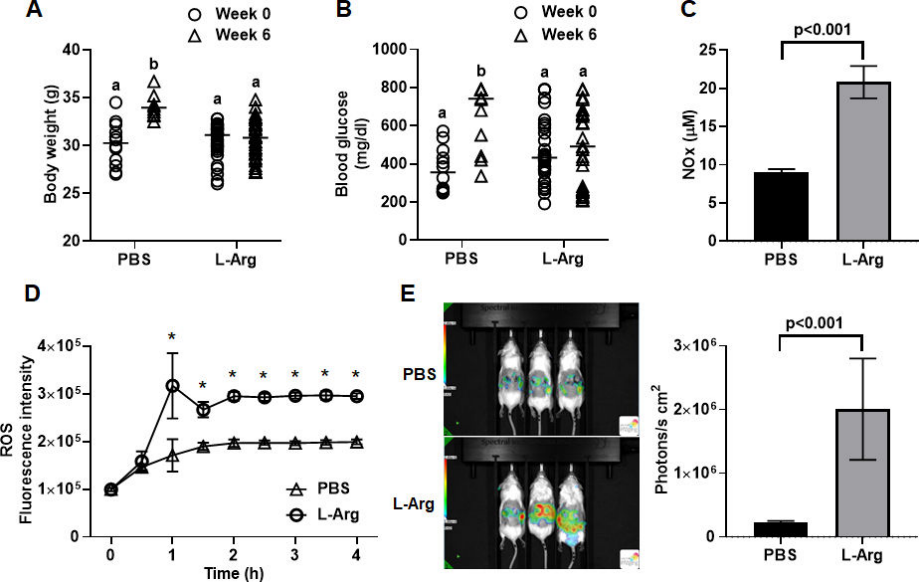
Dietary L-Arg supplementation maintains body weight and serum glucose and increases ROS and nitrate production in diabetic TALLYHO/JngJ mice. (**A**) Body weight and (**B**) serum glucose level in diabetic TALLYHO/JngJ mice before and after gavage with PBS (*n* = 12) or L-Arg for 6 weeks (*n* = 35). (**C**) Serum nitrite/nitrate (NOx) level and (**D**) ROS response of bone marrow-derived neutrophils from diabetic TALLYHO/JngJ mice after gavage with PBS or L-Arg for 6 weeks. (**E**) Representative images of *in vivo* ROS/RNS response to pressure wound visualized by L-012 luminol. The alphabet indicates statistical significance determined by the Mann-Whitney test (*P* < 0.0001). The asterisk indicates statistical significance determined by the Student’s *t*-test. *P < 0.01.

In mammalian hosts, L-arginine (L-Arg) is the only and unique substrate for synthesizing nitric oxide (NO) by inducible nitric oxide synthases. The NO produced from L-Arg predominantly reacts with oxidized hemoglobin and is converted into nitrate (29, 30). In addition, as a defense mechanism against nitrosative stress, *S. aureus* produces flavohemoprotein (Hmp), which oxidizes NO into nitrate (31, 32). These results suggest that dietary supplementation of L-Arg may indirectly provide *S. aureus* with nitrate. To test this possibility, we gavaged diabetic TALLYHO/JngJ mice with L-Arg (9 mg/day) or PBS (vehicle control) for 6 weeks. The L-Arg dosage was based on a study demonstrating that a long-term safe level of dietary L-Arg supplementation is at least 30 g/day in adult humans (33). While the body weight and serum glucose levels in animals fed with PBS were continuously elevated, these were not significantly changed in animals fed with L-Arg, albeit they remained within the ranges of obesity and diabetes (Fig. 5A and B). Serum nitrate/nitrate levels were significantly higher in animals fed with L-Arg than animals fed with PBS (Fig. 5C). Tert-butyl hydroperoxide induced significantly higher reactive oxygen species (ROS) response from bone marrow-derived neutrophils isolated from animals fed with L-Arg than those from animals fed with PBS (Fig. 5D). Consistently, *in vivo* ROS response to pressure wound stimuli was also significantly higher in animals fed with L-Arg than PBS (Fig. 5E). These results indicated that dietary supplementation of L-Arg significantly increased NO synthesis and nitrate/nitrite levels in the mice.

**Fig 6:**
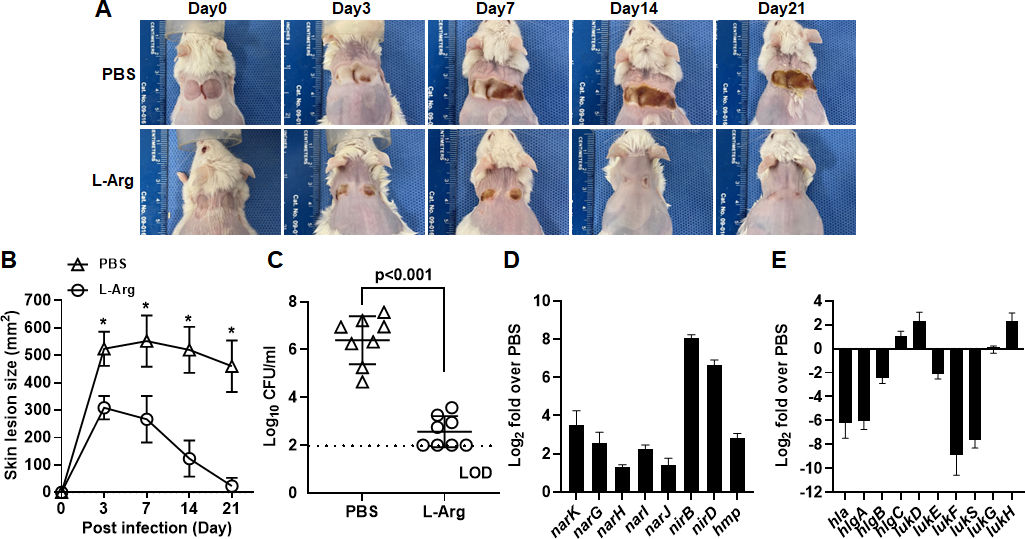
Dietary L-Arg supplementation significantly decreases the severity of disease caused by *S. aureus*-infected pressure wound in diabetic TALLYHO/JngJ mice. (**A**) Representative images of diabetic TALLYHO/JngJ mice gavaged with L-Arg or PBS subcutaneously infected with 1 × 10^7^ of *S. aureus* LAC strain to the ischemic pressure wound (*n* = 8). Progress of wound healing was monitored for 21 days. (**B**) Analysis of areas of wound measured using the SilhouetteStar camera. (**C**) Analysis of bacterial burden of infected pressure wound at day 21 post-infection. (**D and E**) qRT-PCR analysis of (**D**) TCRS and (**E**) virulence factors of infected pressure wounds of L-Arg fed over PBS fed diabetic TALLYHO/JngJ mice at day 7 post-infection. Data are presented as the mean ± standard deviation of three independent experiments. The asterisk indicates statistical significance evaluated by Student’s *t*-test. *P < 0.01. LOD, limit of detection.

To test whether increased nitrate by L-Arg supplementation provides a therapeutic benefit, diabetic TALLYHO/JngJ mice fed with L-Arg or PBS for 6 weeks were infected with *S. aureus* LAC in pressure wounds. Animals fed with L-Arg developed significantly smaller skin lesions (*P* < 0.001) which were completed healed by day 7 post-infection, compared to animals fed with PBS, which remained unhealed with severe tissue necrosis at day 21 post-infection (Fig. 6A and B). Microbiological analysis showed that bacterial burden was significantly less in animals fed with L-Arg than those fed with PBS (Fig. 6C). For qRT-PCR analysis on tissue homogenates, animals were humanely euthanized on day 7 post-infection at which active infection was occurring. Results showed that transcription of genes related to nitrate respiration and the *hmp* gene was highly increased (Fig. 6D), while transcription of many staphylococcal cytotoxin genes was decreased in animals fed with L-Arg (Fig. 6E), compared to animals fed with PBS. Combined, these results suggest that increased nitrate levels by L-Arg supplementation induced anaerobic nitrate respiration in *S. aureus*, which suppressed expression of staphylococcal virulence factors that attenuated the virulence of *S. aureus* within hypoxic pressure wounds of diabetic TALLYHO/JngJ mice.

## DISCUSSION

DFUs result from hyperglycemia causing peripheral neuropathy and microvascular occlusion progress, leading to chronic unhealed ischemic tissue necrosis vulnerable to secondary infections, most commonly by *S. aureus*. For successful pathogenesis, *S. aureus* must adapt to the unique metabolic conditions of DFUs, including hyperglycemia and ischemic hypoxia. Although the effect of hyperglycemia on the pathogenesis of *S. aureus* is well documented, it is relatively less known how hypoxia affects the virulence of *S. aureus*, especially *in vivo*. In this study, we reproduced ischemic hypoxic conditions of clinical DFUs using a pressure wound model in diabetic TALLYHO/JngJ mice. Male TALLYHO/JngJ mice have polygenetic etiology to spontaneously develop hyperglycemia, hyperinsulinemia, hyperlipidemia, moderate obesity, and enlargement of the islets of Langerhans at the age of 12–14 weeks that mimics many characteristics of human non-insulin-dependent T2DM (34). Our results demonstrate that *S. aureus* infections in diabetic hypoxic pressure wounds result in severe chronic tissue necrosis resembling the human clinical *S. aureus-*infected DFUs. These results suggest that hypoxic tissue conditions in DFUs play an important role in the pathogenesis of *S. aureus*, and a pressure wound model in diabetic TALLYHO/JngJ mice serves as an important animal model. The validity of this model will be further warranted by testing more diverse lineages of clinical *S. aureus* isolated from human DFUs.

*S. aureus* possesses several TCRS to respond to hypoxic conditions. Impaired respiration increases menaquinone accumulation, which activates SrrAB to trigger upregulation of NO resistance genes and promotes biofilm formation and cytotoxin production in an AgrA-dependent manner (26, 35–38). NreBC is another TCRS involved in responding to nitrogen. NreB is a sensor histidine kinase with nitrate reductase forming the Fe-S cluster at the C-terminus (39). Hypoxic conditions induce the reduction of the Fe-S cluster, leading to dimerization of NreB and activation of the NreC response regulator, while aerobic conditions induce oxidation of the Fe-S cluster, inhibiting dimerization of NreB for inactivation (39, 40). Inactivation of NreB renders *S. aureus* unable to reduce nitrate as required for anaerobic nitrate respiration, forcing *S. aureus* metabolic flux to fermentative growth for survival (41, 42). Our results showed that *S. aureus-*infected diabetic hypoxic pressure wounds induced transcriptional increase of genes related to anaerobic respiration, including *srrAB* and *nreBC*, and anaerobic nitrate respiration despite increased *S. aureus* virulence factor expression, which is a characteristic of anaerobic fermentative growth. These results suggest that *S. aureus* might be forced to undergo anaerobic fermentative growth due to the lack of sufficient TEA in diabetic hypoxic pressure wounds. This was supported by our *in vivo* findings showing that L-Arg supplementation increases NO synthesis and subsequently increases nitrate/nitrite concentration, which could serve as a TEA for *S. aureus* anaerobic nitrate respiration, leading to decreased expression of virulence factors that resulted in attenuation of *S. aureus* virulence in hypoxic pressure wounds. These findings suggest that antagonizing fermentative growth or augmenting anaerobic respiration might be a metabolic target to attenuate the virulence of *S. aureus* in DFU. This would decrease the need for stronger antibiotic therapy or might even eliminate the need for antibiotic therapy in DFU completely.

One may argue that decreased severity of *S. aureus* infection might be attributed to the increase in NO production by L-Arg supplementation as NO is an important mediator of host innate immunity with potent anti-microbial effects. However, it is noteworthy that *S. aureus* is resistant to very high concentrations of NO due to several NO detoxifying mechanisms such as L-lactate dehydrogenase (Ldh1) balancing redox potential when respiration is inhibited (37, 38) and expression of flavohemoprotein, Hmp, reducing NO to nitrate (31, 32). In fact, transcription of *hmp* was highly increased in our *S. aureus* infection in animals fed with L-Arg. Thus, increased NO production by L-Arg supplementation in the host could not fully but may partially explain the decreased virulence of *S. aureus* in animals fed with L-Arg.

In addition to modulating *S. aureus* pathogenesis, L-Arg supplementation may provide additional benefits for the host. There is growing clinical evidence indicating that long-term dietary L-Arg supplementation can reduce the onset of T2DM by improving insulin secretion and sensitivity, as well as reducing obesity and hyperglycemia (43), as seen in our animals fed with L-Arg daily. Metabolism of L-Arg by arginase-1 provides L-proline and polyamines for collagen synthesis and cell proliferation, respectively, which can improve wound healing (44), also as seen in our L-Arg-fed animals. Collectively, these results suggest that L-Arg could be useful as an alternative/adjunctive therapeutic to prevent severe *S. aureus* infections of DFUs.

L-Arg has been marketed for the purpose of enhancing athletic performance and wound healing, and decreasing high blood pressure and diabetes, but has shown mixed results (45). This is at least partially due to its semi-essential nature. Healthy adult individuals normally synthesize enough L-Arg to saturate endothelial NO synthase activity (46). Therefore, the effect of dietary L-Arg supplement in healthy adult individuals might be minimal. However, supplementation may be needed in special medical conditions such as malnutrition, burns, wounds, infections, and diabetes, in which L-Arg levels are lower than in healthy individuals (46–49). Furthermore, a metabolomics study found that asymmetric dimethylarginine (ADMA) is elevated in T2DM patients (50, 51). ADMA is an analog of L-Arg that inhibits NO synthesis that impairs endothelial function, which leads to atherosclerosis. ADMA is a novel risk marker for cardiovascular disease, and L-Arg supplementation has been shown to improve endothelium-mediated vasodilation that counters this arterial stiffening and thus furthers its potential importance as a T2DM therapeutic. Future randomized and long-term controlled clinical trials will be necessary to further validate the beneficial effect of dietary L-Arg and define the cellular and molecular mechanisms.

Treatment for *S. aureus*-infected DFUs has largely relied on antibiotics, offloading affected feet and debridement, resulting in high levels of conservative treatment failure, recurrence of ulcers, and antibiotic resistance. Even when successful, these conservative treatments can be costly and time consuming while also impeding the quality of life for the patient. Thus, our findings indicate that L-Arg supplementation might be a novel and cost-effective alternative approach to prevent severe *S. aureus* infection in DFU and significantly decrease morbidity and mortality of T2DM patients worldwide.

## MATERIALS AND METHODS

### Bacterial strain and culture conditions

*S. aureus* USA 300 LAC strain was obtained from the Network on Antimicrobial Resistance in *Staphylococcus aureus*. This clonal strain of methicillin-resistant *S. aureus* (MRSA) is the most common community-acquired MRSA causing skin and soft-tissue infections, which makes LAC relevant to study DFU infections (25). *S. aureus* LAC chromosomally integrated with the *gfp* gene was generated using a pTH100 plasmid as described previously (52). *S. aureus* LAC strain was cultured at 37°C for 24 hours in BHI-G6P under anaerobic conditions created by the Mitsubishi AnaeroPack system with or without sodium nitrate (NaNO_3_) treatment.

### Pressure wound model

Male TALLYHO/JngJ and SWR/J mice were purchased from the Jackson Laboratory at the age of 8  weeks and were allowed to acclimate for 1 week. Mice were randomly assigned to individually ventilated cages and housed at 20°C–25°C and 40°C–60% humidity with a 12-hour light cycle. Blood (from tail vain) glucose levels were monitored using an AlphaTrak2 monitoring system (Zoetis) to track hyperglycemia. When TALLYHO/JngJ mice spontaneously developed hyperglycemia (blood glucose >300 mg/dL sustained for more than 3 consecutive days), the animals were anesthetized with isoflurane (Vetone) and shaved from the nape of the neck to the tail head on the dorsum, and additional hair was removed with depilatory cream. To induce ischemic pressure wounds, the dorsal skins over the scapulae were pinched up, and two neodymium rare earth magnets (0.375-in. diameter × 0.125 in. thick, 3,466 G; K&J Magnetics) were placed to the pinched skin fold for two cycles of 12 hours on and off (53). Carprofen (2.5–5.0 mg/kg) was administered subcutaneously at induction to reduce pain. Overnight culture of *S. aureus* LAC grown in the BHI-G6P broth was adjusted to 1 × 10^9^ CFU/mL in PBS, and 10 µL of bacterial suspension was subcutaneously injected in the pressure wound or without pressure wound (*n* = 8 mice/group in two independent experiments). The area of the skin lesions was measured daily with a SilhouetteStar camera and accompanying Silhouette Central data analysis software (ARANZ Medical). Mice were humanely euthanized via CO_2_ inhalation on day 7 post-infection for RNA analyses or day 21 post-infection for bacterial count analyses.

### Antibiotic resistance assay

*S. aureus* LAC was cultured in BHI-G6P broth supplemented with a twofold serial dilution of vancomycin (0–16 µg/mL) or cefoxitin (0–32 µg/mL) at 37°C under either aerobic/anaerobic conditions or ±NaNO_3_ treatment by minimum inhibitory concentration (MIC) assays. The MIC of each antibiotic was measured at OD_600_ for 24 hours by a Cytation5 microplate reader (BioTek Instruments, Inc.).

### Biofilm assay

Sterile two-well glass chamber slides (Celltreat) were treated with 20% human serum in sterile carbonate-bicarbonate buffer (Sigma) for overnight at 4°C. *S. aureus* LAC strain chromosomally integrated with the *gfp* gene was inoculated into BHI broth supplemented with G6P (and incubated at 37°C for 72 hours under hypoxic/anaerobic conditions or ±NaNO_3_ treatment. Plates were washed with PBS and placed on a Mixer HC plate shaker (USA Scientific) at 500 rpm for 3 minutes twice to remove non-adherent cells. The two- and three-dimensional images and thickness of biofilms were analyzed using an Axiovert 200 M Inverted Fluorescence Microscope (Zeiss).

### Hemolysis assay

The supernatants of *S. aureus* LAC cultures were collected and filtered with polyethersulfone (PES) 0.45-µm syringe filters (CellTreat). Fresh whole human blood was collected in an EDTA-containing vacutainer tube (Monoject Covidien). After centrifugation at 1,300 rpm for 3 minutes, RBCs were collected and plasma was discarded, washed twice with PBS, and diluted to a final 2% RBC in PBS solution. Fifty microliters of RBC and 50 µL of exoprotein samples were added to each well in a 96-well cell culture V bottom plate (Cell Treat) and incubated at 37°C with 5% CO_2_ for 1 hour. Sterile medium and LukF/S (1 µg of each, IBT Bioservices) were used as negative and positive controls, respectively. Hemolysis was analyzed by measuring the optical density at 540 nm using a Cytation 5 reader (BioTek Instruments, Inc.).

### Quantitative real-time PCR analysis

Total RNA was extracted from bacteria or infected mouse tissues using a RNeasy extraction kit according to the manufacturer’s instructions (Qiagen). A total of 100-ng RNA was measured by NanoDrop OneC Microvolume UV-Vis Spectrophotometer (Thermo Scientific) and used to perform qRT-PCR using a Superscript one-step qRT-PCR kit (Invitrogen). The primers are listed in Table S1. The data were normalized by calculating the threshold cycle (CT) of the target minus the CT of the internal control (gyrA for bacterial RNA) (ΔCT). Relative quantification of the target gene was determined by the comparative CT method (ΔΔCT) (either aerobic vs hypoxic or −NaNO_3_ vs + NaNO_3_).

### Analysis of phosphorylation of TCRS using Phos-Tag

The AgrA-FLAG, SaeR-FLAG, or HptR-FLAG gene was amplified from *S. aureus* LAC chromosomal DNA with primers listed in Table S1 and cloned between the HpaI/EcoRI sites in the tetracycline inducible expression vector, pRMC2. The plasmids were constructed in *Escherichia coli* DH5α and then transformed to *S. aureus* RN4220 prior to electroporation into *S. aureus* LAC, as described previously (54). Constructed plasmids were confirmed by PCR and sequencing. Constructed *S. aureus* strains were selected on BHI agar containing chloramphenicol (10 µg/mL) at 37°C. Transformed *S. aureus* LAC strains were cultured in BHI-G6P broth containing chloramphenicol (5 µg/mL) and anhydrotetracycline (10 µg/mL) with or without supplementation of 2-mM NaNO_3_ for 6 hours at 37°C under hypoxic condition entering mid-log growth phase. Bacterial cells were disrupted by bead beating using FastPrep-24 (MP Biomedicals), and then the lysates were centrifuged at 11,000 × *g* for 10 minutes at 4°C. A total of 5 µg of protein was loaded onto 12.5% SuperSep Phos-tag Precast Gels (Wako Chemicals). The separated proteins were blotted onto polyvinylidene difluoride membranes using Power Blotter–Semi-dry Transfer System (Invitrogen) according to the manufacturer’s instructions. The membrane was blocked with 5% skim milk for 16 hours at 4°C and immunoblotted with mouse anti-FLAG M2-peroxidase monoclonal antibody (Sigma) for 1 hour at room temperature. After washing a membrane with TBS containing 0.05% Tween 20, bound antibody was detected with SuperSignal West Pico PLUS Chemiluminescent Substrate (Thermo Scientific) and ChemiDoc MP Imaging System (Bio-Rad).

### Dietary L-arginine supplementation

Diabetic TALLYHO/JngJ mice (*n* = 35) were gavaged with 30 µL of L-arginine in PBS (30% [wt/vol], Sigma-Aldrich) once daily (9 mg L-Arg/mouse/day) and changes in body weight and serum glucose levels were monitored weekly. As a control, the same cohort of animals (*n* = 12) were gavaged with 30 µL of PBS. After feeding L-Arg for 6 weeks, a pressure wound infection was performed as described above.

After 6 weeks of feeding but before infection, serum samples were collected via retro-orbital bleeding, and serum nitrate/nitrite concentration was measured using the Nitrate/Nitrite Colorimetric Assay Kit (Cayman Chemical) according to the manufacturer’s instructions. Briefly, serum samples were filtered through 10-kDa molecular weight cutoff filters (Amicon Ultra, Millipore). All nitrates in the serum samples were converted to nitrite by nitrate reductase treatment. Total nitrite concentration was detected using Griess reagents with absorbance measured at 540 nm on a Cytation 5 plate reader (BioTek Instruments, Inc.). Sample values were plotted against a nitrite standard curve to determine concentration.

To measure ROS response from bone marrow-derived neutrophils, neutrophils were isolated from bone marrow as described previously from non-infected animals (55). ROS was detected using the 2,7-dichlorofluorescein diacetate (DCFDA) cellular ROS detection assay kit (Abcam) according to the manufacturer’s instructions. In brief, bone marrow-derived neutrophils were resuspended in Krebs-Ringer phosphate glucose solution (Thermo Fisher Scientific) containing 20 µM DCFDA. Cells were then stimulated with 50 µM tert-butyl hydroperoxide. Fluorescence was measured at 485 nm/535 nm with a Cytation 5 plate reader (BioTek Instruments, Inc.) for 4 hours.

To measure *in vivo* ROS response from a pressure wound, a ROS-sensing probe, L-012 luminol (10 mg/mL, Labchem Wako) stock solution, was prepared in normal saline and intraperitoneally injected with 10 mL/kg of stock solution to achieve a final dosage of 100 mg/kg (56). After injection, animals were imaged using the Ami HTX (Spectral Instruments Imaging, Tucson, AZ, USA). Images were processed and analyzed using Aura Software version 4.0.8 (Spectral Instruments Imaging). Data were presented as total flux in photons per second per region of interest (photons/s/cm^2^).

### Statistical analysis

The statistical significance of data for the biofilm, hemolysis, growth curves, NOx, MIC tests, wound measurements were analyzed by Student *t*-test and Mann-Whitney test using GraphPad Prism version 9.4.1 (GraphPad, San Diego, CA,USA).

## Data Availability

All data generated or analyzed during this study are included in the main text and supplemental table. Additional data related to this article are available from the corresponding author upon reasonable request.

## SUPPLEMENTAL MATERIAL

TABLE S1 msphere.00774-23-s0001.docx A list of primers used in the study.

ASM does not own the copyrights to Supplemental Material that may be linked to, or accessed through, an article. The authors have granted ASM a non-exclusive, world-wide license to publish the Supplemental Material files. Please contact the corresponding author directly for reuse.
