# Supplementary material for: L-arginine supplementation abrogates hypoxia-induced virulence of Staphylococcus aureus in a murine diabetic pressure wound model
Source: mSphere. 2024 Mar 1;9(3):e00774-23. doi: 10.1128/msphere.00774-23 (PMC10964415; doi:10.1128/msphere.00774-23)
Supplement: TABLE S1 — A list of primers used in the study. [file msphere.00774-23-s0001.docx]

Table 1. A list of primers used in the study.

| **Primer** | **Sequence (5’-3’)** |
| --- | --- |
| RT_icaAF | TTGGTGCAGTTACAGGTAATCCTAGA |
| RT_icaAR | CCAGTAGCCAACGTCGACAA |
| RT_icaBF | CACATACCCACGATTTGCAT |
| RT_icaBR | TCGGAGTGACTGCTTTTTCC |
| RT_icaCF | CTTGGGTATTTGCACGCATT |
| RT_icaCR | GCAATATCATGCCGACACCT |
| RT_narGF | CACAACGCGCCGACTAGA |
| RT_narGR | TTGGTTCCCAATTGGTCCAT |
| RT_narHF | CGGCGAGCATTTTGGAGAT |
| RT_narHR | TGACCAGTTTTCCCTGAACCTACT |
| RT_narIF | TGTTTTTTGGACACGTAGTTGGA |
| RT_narIR | GCTCAGACACACCCACTGCAT |
| RT_narJF | CAAAAAGAACGTGGGCAAAT |
| RT_narJF | AGTGCATTCGCCATCACATA |
| RT_narKF | TTGTTGGCGCTAAATGGGTAT |
| RT_narKR | CAAGGAAAAATCCTGAAGCCATTA |
| RT_ddlF | CATGGCCAGGTGAAGTCGTA |
| RT_ddlR | TGCCTCTAATGCCATATTTCTAAGC |
| RT_pbp2F | CAACTGAAGACAATCGTTTCTACGA |
| RT_pbp2R | ACCAATTGCACCGAATAAACGT |
| RT_pbp3F | AAGAAATGAAATACACAACGGACAAA |
| RT_pbp3R | GCGAGCGCCAGGATTTAAC |
| RT_pbp4F | ACAGTGACGTAACCCCTGTACAAG |
| RT_pbp4R | CGTATGCAGCCGACAAACC |
| RT_arlSF | AAAAAAGACCCAGCAGTATTAGAAGAA |
| RT_arlSR | CGCGAGCGAATTTCATCATT |
| RT_arlRF | CTTGATTACGGTGCAGACGATTATA |
| RT_arlRR | ACCGTTGACATCGATAATATCCTTT |
| RT_nirBF | GGCTGATGGACGCGAGAT |
| RT_nirBR | TGGTCGTATACCTACTGCCATAACA |
| RT_nirDF | GCAAAATTCATGCGATTCACAA |
| RT_nirDR | TCACTGTCCCTTCAGACAATGGT |
| RT_nreBF | ATCACAAATTGAACAGCAAGAGAAA |
| RT_nreBR | TGCGTCTTCCAAAAGCTTAGTTG |
| RT_nreCF | GCGTCGAAGCTTACCAAAAA |
| RT_nreCR | ACCATTACGCAACACATGGA |
| RT_srrAF | CGTCAAACCATTTTCACCAAGA |
| RT_srrAR | GCGATGTGCATCATTATCTATTTCTAA |
| RT_srrBF | GTACAGGCATTGCACCAGAA |
| RT_srrBR | GCCTTTCCCTAATTCGCTTT |
| RT_hptSF | ACGTCGCTTGCGTGATATTCA |
| RT_hptSR | TGGACCACAGTTTGTGCTAATT |
| RT_hptRF | AAGCCGGCATTCAACATCATGT |
| RT_hptRR | TACGGCCGTTCTGTGATTGTT |
| RT_agrAF | TTTGCACAAGACCCAACTGG |
| RT_agrAR | ATCTGGGACGAAATAGTCTCGTG |
| RT_agrCF | ATGCGCAAGTTCCGTCATG |
| RT_agrCR | TTCTGAAAGTGTCGTTAAGATATTGACA |
| RT_saeSF | ATCATTATTGGCGTCGTATCGAG |
| RT_saeSR | GTCAAAGTTAGTGTCATATGGCCG |
| RT_saeRF | ATGACCCACTTACTGATCGTGGA |
| RT_saeRR | CCGCTAGTTGTCGTTGTTACTTTG |
| RT_hlaF | GTTTAGCCTGGCCTTCAGCC |
| RT_hlaR | TTTTGTATCAATCGAATTTCTTGGAT |
| RT_hlgAF | TTTGCACAAGACCCAACTGG |
| RT_hlgAF | ATCTGGGACGAAATAGTCTCGTG |
| RT_hlgBF | TCACACAGACAAGATGGCGC |
| RT_hlgBR | CCTGCCCAGTAGAAGCCATTC |
| RT_hlgCF | AAAAGATCAACGCATTATGGCA |
| RT_hlgCF | TTCCAATTGACCTCGTATTTCACA |
| RT_lukFF | CGAAAACAAAACGCTGCAAA |
| RT_lukFR | TTACCTATCCAGTGAAGTTGATTCCA |
| RT_lukSF | AGCTGCAACATTGTCGTTAGGA |
| RT_lukSR | CCTCAGCGCCATCACCA |
| RT_lukDF | GGATCAAAATCATTTTCAGAAACGA |
| RT_lukDR | TCAACACCCCAGCCAATTG |
| RT_lukEF | CAAAGTCCAAATGGTCCAACAG |
| RT_lukER | GTGATAAACGATGGATTAAAGCCA |
| RT_lukGF | TTTGCACCAAAAAATCAGGATG |
| RT_lukGF | TTTCCAGTTAAGCCTCCACGAT |
| RT_lukHF | CAAAAGGTATTGGACGAACTTCATC |
| RT_lukHR | CCAGTTATTATTTTTACCGCTGGC |
| RT_hmpF | TGACTTTAGTGAATTTACACCAGG |
| RT_hmpR | CGTTTAACGCCAAAAGTTAAATGG |
| RT_gyrAF | CGTGAAGGTGACGAAGTTGTAGG |
| RT_gyrAR | TAACTGGCGTACGTTTACCATAAC |
| AgrAFlagR_EcoRI | AATTGAATTCTTATTTATCGTCATCATCCTTGTAGTCTTTATC |
|  | GTCGTCATCTTTATAATCTTTGTCATCATCATCTTTGTAATCTATTTTTTTAACGTTTCTCACCGA |
| AgrAF_HpaI | AATTGTTAACCATAAGGATGTGAATGTATGAAAATTTTC |
| HptRFlagR_EcoRI | AATTGAATTCTTATTTATCGTCATCATCCTTGTAGTCTTTATC |
|  | GTCGTCATCTTTATAATCTTTGTCATCATCATCTTTGTAATCTTTTGCTTGCTTACAATAATCACT |
| HptRF_HpaI | AATTGTTAACTTCAAGGGGGAATGTAGATGTTTAAGGT |
| SaeRFlagR_EcoRI | AATTGAATTCTTATTTATCGTCATCATCCTTGTAGTCTTTATC |
|  | GTCGTCATCTTTATAATCTTTGTCATCATCATCTTTGTAATCTCGGCTCCTTTCAAATTTATATC |
| SaeRF_HpaI | AATTGTTAACCAGAGGTGAAAAAATAGATGACCCACTT |
